# Six3 regulates optic nerve development via multiple mechanisms

**DOI:** 10.1038/srep20267

**Published:** 2016-01-29

**Authors:** Anat Samuel, Ariel M. Rubinstein, Tehila T. Azar, Zohar Ben-Moshe Livne, Seok-Hyung Kim, Adi Inbal

**Affiliations:** 1 Department of Medical Neurobiology, Institute for Medical Research - Israel-Canada, The Hebrew University-Hadassah Medical School, Jerusalem, Israel; 2 Department of Neurobiology, The George S. Wise Faculty of Life Sciences and Sagol School of Neuroscience, Tel Aviv University, Tel Aviv, Israel; 3 Department of Biological Sciences, Vanderbilt University, Nashville, Tennessee, USA

## Abstract

Malformations of the optic nerve lead to reduced vision or even blindness. During optic nerve development, retinal ganglion cell (RGC) axons navigate across the retina, exit the eye to the optic stalk (OS), and cross the diencephalon midline at the optic chiasm en route to their brain targets. Many signalling molecules have been implicated in guiding various steps of optic nerve pathfinding, however much less is known about transcription factors regulating this process. Here we show that in zebrafish, reduced function of transcription factor Six3 results in optic nerve hypoplasia and a wide repertoire of RGC axon pathfinding errors. These abnormalities are caused by multiple mechanisms, including abnormal eye and OS patterning and morphogenesis, abnormal expression of signalling molecules both in RGCs and in their environment and anatomical deficiency in the diencephalic preoptic area, where the optic chiasm normally forms. Our findings reveal new roles for Six3 in eye development and are consistent with known phenotypes of reduced *SIX3* function in humans. Hence, the new zebrafish model for Six3 loss of function furthers our understanding of the mechanisms governing optic nerve development and Six3-mediated eye and forebrain malformations.

The retina comprises five neuronal cell types that are organized in three layers of cell bodies. Of these, only retinal ganglion cells (RGCs), whose cell bodies are located in the ganglion cell layer (GCL), send axons via the optic nerve that relay information outside of the retina, to the optic tectum in non-mammalian vertebrates and to the superior colliculus (SC) in mammals.

The journey of RGC axons is complex. As RGCs differentiate, their axons navigate to the optic disc (optic nerve head), fasciculate and exit the eye as the optic nerve^1^,^2^. After leaving the eye, axons navigate to the ventral diencephalon midline where they form the optic chiasm in the preoptic area (POA) with axons from the contralateral eye by crossing to the contralateral optic tract on their way to the tectum/SC. In animals with monocular vision such as zebrafish, all RGC axons cross the midline^3^. Innervation of the tectum/SC forms a retinotopic map, such that axons from cells located adjacently in the GCL project to adjacent locations in the tectum/SC^1^,^4^.

In zebrafish, forward genetic screens have identified several types of RGC axon misrouting phenotypes. Pathfinding errors include abnormal navigation within the eye, failure to cross the midline coupled with innervation of the ipsilateral tectum, and ectopic innervation of forebrain structures^5^,^6^,^7^,^8^. Many mechanisms, both cell-autonomous and non cell-autonomous, underlie the various RGC pathfinding errors and include abnormal eye and optic stalk (OS) patterning, reduced expression of receptors such as *robo2* and *cxcr4b* in RGCs or their ligands in the environment where axons navigate^9^,^10^, failure to develop RGC pioneers^11^ and abnormal patterning of the ventral diencephalon midline^12^. However, little is known about how expression of the signalling pathway components is regulated and relatively few transcription factors have been implicated in regulation of optic nerve pathfinding.

Six3 is a transcription factor essential for normal forebrain and eye development. In humans, *SIX3* haploinsufficiency can cause holoprosencephaly (HPE), the most common forebrain malformation characterized by midline deficiencies of brain and facial structures as well as a wide spectrum of eye malformations such as cyclopia, anophthalmia, microphthalmia and coloboma^13^,^14^,^15^. In current models of Six3 loss of function, eye formation is severely abrogated from early stages. Mice homozygous for *Six3* null mutation lack all forebrain structures including eyes^16^ and conditional removal of *Six3* from the mouse eye field resulted in arrested neural retina specification^17^. In medaka, reduced Six3 function resulted in reduced eye tissues and cyclopia was also observed^18^. In zebrafish there are three *six3*-related genes, *six3a*, *six3b* and *six7*^19^,^20^. The combined loss of zebrafish *six3b* and *six7* function resulted in anophthalmia or microphthalmia^21^. Hence, to date it has been difficult to study the roles of Six3 in later stages of eye development, when Six3 is expected to function based on its expression, which is initially observed throughout the developing eye at optic vesicle stage, becomes largely limited to the OS and ventral eye at optic cup stage^19^,^20^,^22^,^23^,^24^, and is limited to the retina inner nuclear layer and RGCs after neurogenesis^25^,^26^,^27^.

Here we describe a new zebrafish model for Six3 loss of function that reveals new roles for Six3 affecting later eye development. We show that zebrafish embryos with reduced Six3a and no Six3b functions have malformed eyes with large optic disc colobomas. The optic nerve in these embryos is hypoplastic and severely defasciculated and RGC axons make multiple pathfinding errors, both in eye and in forebrain. These errors appear to result from multiple mechanisms, including abnormal eye patterning, failure of OS differentiation, abnormal differentiation of RGCs and deficiencies in the diencephalic midline. Altogether, our findings show that Six3 is required for normal RGC and optic nerve development and identify *six3a;six3b* double mutants as a useful model for revealing new roles for Six3 in eye development.

## Results

### *six3a;six3b*-deficient embryos

**Figure 1:**
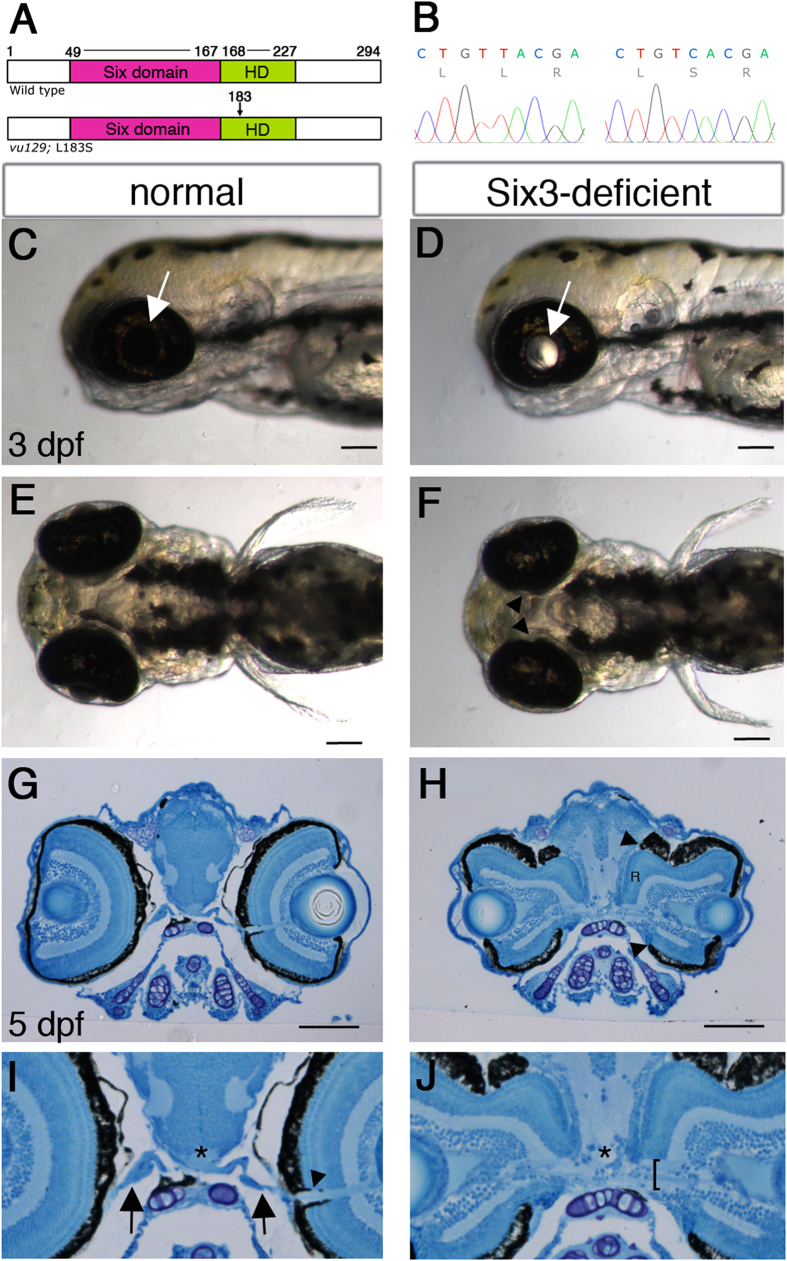
Optic disc coloboma and optic nerve malformations in *six3a;six3b* double mutant embryos. (**A**) Schematic presentation of WT Six3a and Six3a^L183S^ proteins. Amino acid numbers are depicted and location of L183S in the homeodomain is marked with an arrow. HD, homeodomain. (**B**) Chromatograms showing the sequence difference between WT (left) and mutant (right) *six3a*. (**C–F**) Lateral views (**C,D**) and ventral views (**E,F**) of 3 dpf live normal (**C,E**) and Six3-deficient (**D,F**) embryos. Arrows in (**C,D**) point at lenses, arrowheads in F point at the medial, incomplete RPE. (**G–J**) Low (**G,H**) and higher (**I,J**) magnifications of histological transverse sections through forebrains of normal (**G,I**) and Six3-deficient (**H,J**) 5 dpf embryos. “R” in (**H**) marks ectopic retinal tissue of one eye. Arrowhead and arrows in I point at the optic nerve inside and outside the eye, respectively, which cannot be clearly seen in the double mutant; asterisks mark location of the optic chiasm; bracket in J marks an apparently defasciculated optic nerve. (**C,D,G–J**) dorsal is up, (**C–F**) anterior is to the left. Scale bars are 50 μm.

To identify new roles for Six3 in embryonic development we screened for mutations in *six3a* by TILLING^28^,^29^. From all identified mutations, only the *vu129* allele, in which a T to C transition changes a highly conserved Leucine in position 183 in the homeodomain to Serine (L183S) (Fig. 1A,B), caused a severe reduction in Six3a activity, as tested by *six3a* misexpression (Supplementary Fig. S1). These results identify *six3a*^*vu129*^ as a strong hypomorphic allele.

As both *six3a*^*vu129*^ and *six3b*^*vu87*^ homozygous mutants appear normal during embryonic and early larval stages^21^, we examined whether there was redundancy between these genes by generating double mutant embryos. We intercrossed double heterozygotes for *six3a*^*vu129*^ and *six3b*^*vu87*^ and examined their progeny, which appeared normal until 2 days post-fertilization (dpf). However, at 2 dpf, and more prominently at 3 dpf, light could be seen passing through the lenses in some embryos, whose eyes also appeared slightly smaller (Fig. 1D). The passage of light suggested a defect in the retinal pigmented epithelium (RPE) layer, which covers the neural retina, and indeed, the medial region of the RPE appeared incomplete (Fig. 1F). These embryos died around 2 weeks post-fertilization. Genotyping revealed that affected embryos were almost strictly double mutants, carrying four mutant *six3* alleles (n = 22/25), and few (n = 3/25) carried three mutant alleles. We hereafter refer to double mutants as *six3a;six3b* double mutants or Six3-deficient embryos.

To better understand the eye phenotype we performed histological sections at 5 dpf, which revealed large colobomas in the optic disc region with ectopic retinal tissue protruding towards the brain (Fig. 1H). Additionally, optic nerve axons, which normally form a single bundle that exits the eye at the optic disc region (Fig. 1G,I), appeared severely defasciculated (Fig. 1H,J) and the optic chiasm could not be detected (Fig. 1I,J).

Together, these results show that reduced Six3 function through partial loss of Six3a and complete loss of Six3b activities causes abnormal eye and optic nerve morphogenesis.

### Multiple optic nerve abnormalities in Six3-deficient embryos

**Figure 2:**
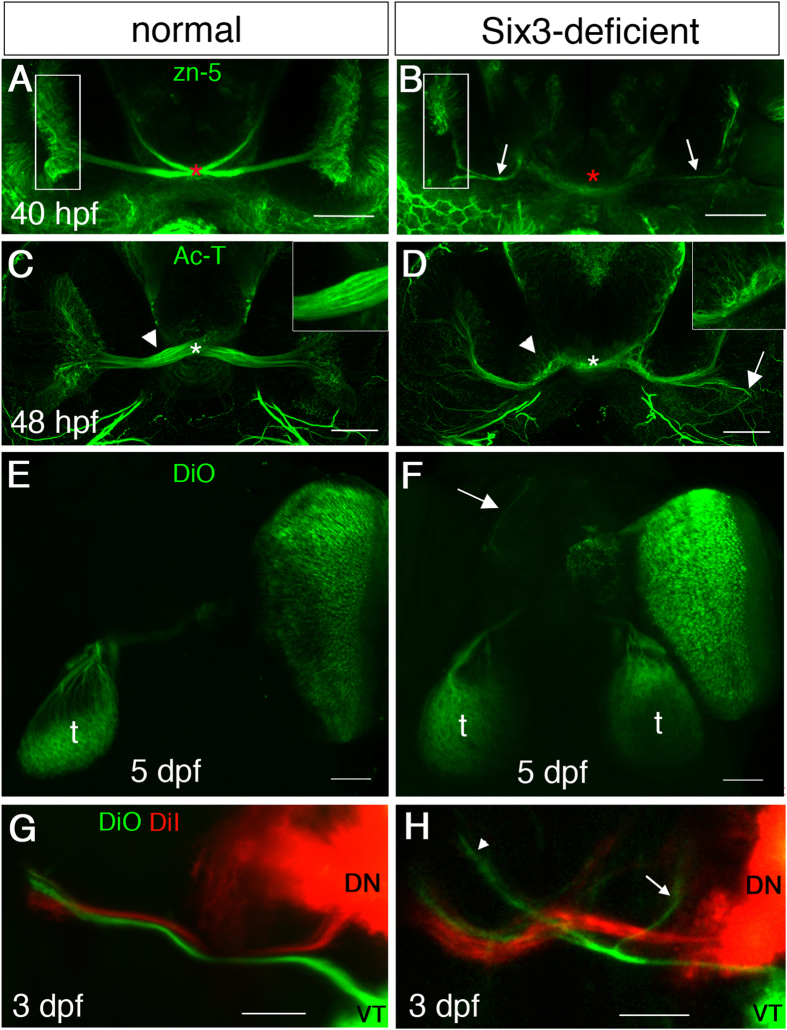
Optic nerve misrouting in Six3-deficient embryos. (**A–H**) Maximum projection confocal images of labelled RGC axons in WT (**A,C,E,G**) and Six3-deficient (**B,D,F,H**) embryos. (**A,B**) zn-5 immunohistochemistry at 40 hpf. Asterisk in A marks the optic chiasm and in (**B**) where the optic chiasm should be. Arrows in B point at RGC axons. Rectangles in (**A,B**) mark the GCL of one eye. (**C,D**) Ac-T immunohistochemistry at 48 hpf. Asterisks in (**C,D**) mark location of the optic chiasm. Arrow in (**D**) points at a region where RGC axons are misrouted and fail to exit the eye. Insets in (**C,D**) show higher magnification of the regions at which arrowheads point. (**E,F**) DiO anterograde labelling from a single eye at 5 dpf. Arrow in F points at axons reaching the forebrain. (**G,H**) DiO (green) and DiI (red) labelling of VT and ND RGCs, respectively, at 3 dpf. t, tectum. In (**H**) arrow points at VT axons remaining ipsilaterally and arrowhead points at VT axons misrouted to the forebrain. (**A–D**) are ventral views and E-H are dorsal views. Anterior is up. Scale bars are 50 μm.

We analyzed more closely the effects of Six3 loss of function on development of the optic nerve. In zebrafish, RGCs begin to differentiate around 28 hours post-fertilization (hpf)^30^,^31^, axons exit the eye around 32 hpf, cross the chiasm at 34–36 hpf and fully innervate the tectum at 72 hpf^32^,^33^. We began by examining the optic nerve at 40 hpf, when axons should have crossed the diencephalic midline. To visualize optic nerve axons we used antibodies against DM-GRASP (zn-5)^34^,^35^ or against acetylated α-tubulin (Ac-T). Compared to normal (Fig. 2A), Six3-deficient embryos had hypoplastic optic nerves, axons failed to reach the midline and fewer RGCs were detected, especially in the ventral retina (Fig. 2B). By 48 hpf, optic nerve axons of Six3-deficient embryos reached the midline but the optic nerve remained hypoplastic and there was no clear structure of an optic chiasm (Fig. 2D). Moreover, some axons appeared to project abnormally inside the eye and many axons appeared to stray from the optic nerve before and in the midline region (Fig. 2D).

To better visualize pathfinding of the optic nerve we used lipophilic dyes for anterograde labelling of RGC axons from one eye, at 3 and 5 dpf. Whereas in WT embryos axons from one eye were always traced only to the contralateral tectum (n = 22) (Fig. 2E), in Six3-deficient embryos axons were found both in the contralateral and ipsilateral tecta (n = 31/35) and also in the telencephalon (n = 34/35) (Fig. 2F, S3).

As the ventral retina appeared to be more strongly affected by Six3 loss of function, we asked whether RGC axons from both dorsal and ventral regions exhibited similar pathfinding defects. We labelled dorsonasal (DN) and ventrotemporal (VT) eye quadrants with DiI and DiO, respectively, and examined RGC axons. In WT embryos (n = 15), all axons from VT and DN retina invariably projected contralaterally (Fig. 2G). In Six3-deficient embryos, axons of VT RGCs made many more pathfinding errors than DN axons. In all examined embryos (n = 17), many VT axons projected to the ipsilateral tectum and/or forebrain, whereas DN axons projected mostly to the contralateral tectum. However, some DN axons also showed aberrant pathfinding and axons projecting to the contralateral tectum were defasciculated (Fig. 2H).

Despite the multiple pathfinding errors, many axons reached either the contralateral or ipsilateral tectum; however, it was unclear whether they formed functional connections. To address this question we performed the visual motor response (VMR) test, which monitors locomotion of larvae that is normally triggered by transitions between light and dark conditions^36^ (see Methods sections for details). We found that although Six3-deficient larvae had a lower baseline activity than WT, they responded to transition from light to dark by a sharp increase of locomotor activity, similarly to WT (Supplementary Fig. S2). When lights were turned on, both WT and Six3-deficient larvae increased their activity, however the response of Six3-deficient larvae was delayed and somewhat slower compared to WT (Supplementary Fig. S2). These results suggest that Six3-deficient larvae can see, indicating functional connections are formed in the tectum. The mechanisms responsible for differences in locomotor behavior compared to WT larvae remain to be determined.

### Six3 is required for normal eye patterning

**Figure 3:**
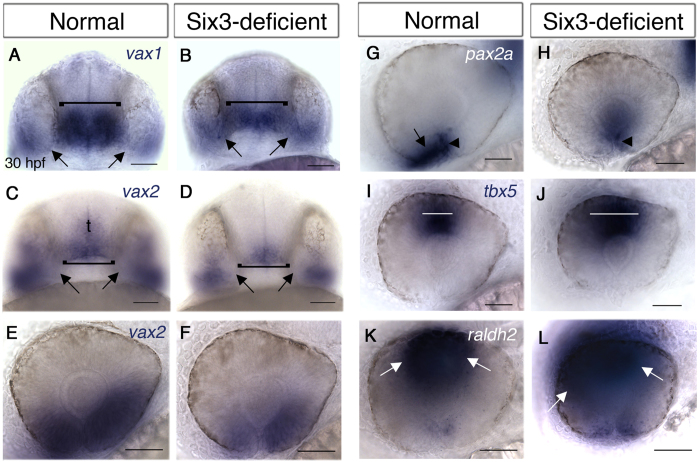
Eyes and optic stalks of Six3-deficient embryos are abnormally patterned. (**A–L**) WISH of 30 hpf normal (**A,C,E,G,I,K**) and Six3-deficient (**B,D,F,H,J,L**) embryos. (**A,B**) *vax1* expression. Brackets marks POAs, arrows point at OSs. (**C–F**) *vax2* expression in the POA, OS and ventral retina. Brackets marks POAs, arrows point at OSs. t, telencephalon. (**G,H**) *pax2a* expression in the optic fissure (arrowheads in G,H) and OS (arrow in **G**, missing in **H**). (**I,J**) *tbx5* expression in dorsal retina. White lines mark the width of the region where high *tbx5* levels are observed. (**K,L**) *raldh2* expression in the dorsal retina. Arrows mark limits of high *raldh2* expression. (**A–D**) are ventral views, anterior is up. E-L are lateral views, anterior to the left, dorsal up. Scale bars are 50 μm.

Next, we addressed potential mechanisms that could cause optic nerve misrouting in Six3-deficient embryos. Optic nerve pathfinding errors and coloboma have been associated with abnormal eye patterning and reduced function of genes required for ventral retina and OS development. In Medaka, reduced Six3 function resulted in abnormal eye patterning with reduced expression of ventral eye genes^18^. Interestingly, the coloboma phenotype of *six3a;six3b* double mutant embryos is highly reminiscent of combined loss of function of transcription factors Vax1 and Vax2^37^,^38^. We therefore examined *vax1* and *vax2* expression at 30 hpf, after initial eye patterning has been completed. At this stage, both *vax1* and *vax2* are normally expressed in the POA, weakly expressed in the OS, and in the ventral retina *vax2* is prominently expressed whereas *vax1* expression is limited to the region of the choroid fissure (Fig. 3A,C,E). In double mutant embryos, *vax1* expression (n = 6) was clearly present in the OS region, and appeared upregulated. Additionally, the OS appeared abnormally large. In the POA, *vax1* expression was reduced and retinal expression appeared comparable to normal (Fig. 3B). *vax2* expression (n = 3) was reduced in the ventral retina, more prominently in the nasal region, almost absent from the OS and reduced in the POA (Fig. 3D,F). These results show that *vax* gene expression is differentially affected by Six3 loss of function, with *vax2* being generally reduced and *vax1* reduced in the POA and upregulated in the OS.

Another transcription factor important for eye and optic nerve morphogenesis is Pax2, whose loss of function results in optic fissure coloboma and similar RGC axon pathfinding defects to Six3-deficient embryos^39^,^40^. At 30 hpf, *pax2a* was expressed in the optic fissure and OS of normal embryos, whereas in double mutants (n = 6) expression in the optic fissure was reduced and was absent from the OS (Fig. 3G,H) (also see below).

In contrast to the reduced expression of ventral genes in double mutants, expression of dorsal eye genes *tbx5* (n = 2) (Fig. 3I,J) and *raldh2* (n = 3) (Fig. 3K,L) was expanded. These results suggest that Six3-deficient eyes are partially dorsalized, an abnormality that can contribute to RGC axon pathfinding defects.

### Optic stalk is induced but fails to differentiate when Six3 function is reduced

**Figure 4:**
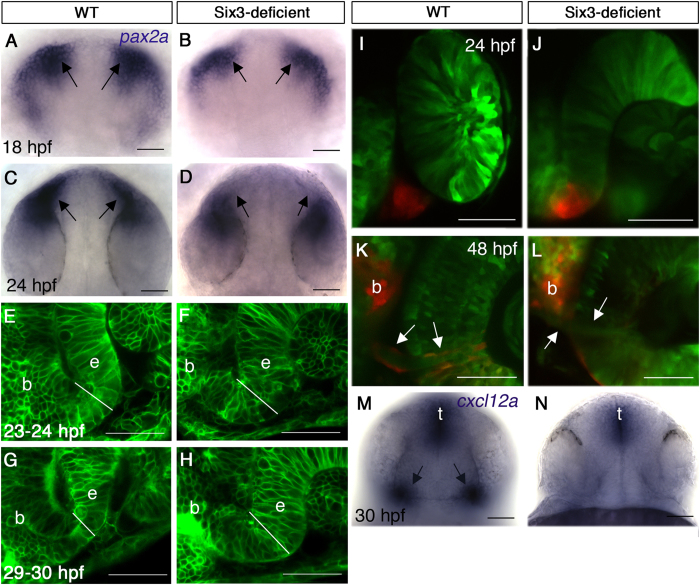
Abnormal optic stalk development in Six3-deficient embryos. (**A–D**) WISH for *pax2a* at 18 hpf (**A,B**) and 24 hpf (**C,D**) in WT (**A,C**) and Six3-deficient embryos (**B,D**). Arrows in (**A–D**) point at the OSs. (**E–H**) Confocal images of OS in live WT (**E,G**) and Six3-deficient (**F,H**) embryos at 23–24 hpf (**E,F**) and 29–30 hpf (**G,H**). Outlines of all cells are highlighted by expression of membrane-tethered GFP. White lines demarcate OS width at the interface with the retina. (**I–L**) Confocal images of *rx3:Kaede* live embryos in WT (**I,K**) and Six3-deficient (**J,L**) backgrounds. The OS was photoconverted in these embryos (red). (**I,J**) Images were captured immediately after photoconversion at 24 hpf. (**K,L**) Images of the same embryos as in I and J, respectively, at 48 hpf. Arrows point at the periphery of the optic nerve, where only in the WT embryo (**K**) photoconverted glial cells are present. (**M,N**) WISH for *cxcl12a* in WT (**M**) and Six3-deficient (**N**) embryos at 30 hpf. Arrows in M point at OS expression of *cxcl12a*. b, brain; e, eye; t, telencephalon. (**A–D**) dorsal views anterior up; (**E–J**) frontolateral views, dorsal up; (**K,L**) ventral views anterior up; (**M,N**) frontal views, dorsal up. Scale bars are 50 μm.

The OS plays a critical role in guiding growing RGC axons and its cells differentiate into astrocytes of the optic nerve. As the OS of Six3-deficient embryos failed to express *pax2a* and appeared abnormal at 30 hpf, we examined whether it was specified in the mutants by labelling for *pax2a* expression during optic cup formation. At 18 hpf (18-somite stage), *pax2a* was expressed in the developing OS of double mutants (n = 3) although in a slightly reduced domain, but this expression disappeared by 24 hpf (n = 6) (Fig. 4A–D). Hence, the OS was specified and the reduction of Six3 activity resulted in failure to maintain *pax2a* OS expression.

Next we examined OS morphogenesis in live embryos in which all cell membranes were labelled by membrane-tethered EGFP. Normally, the OS narrows significantly from 23 hpf to 30 hpf (n = 3–5 embryos for each time point) (Fig. 4E,G). In double mutant embryos, the OS failed to narrow and remained wider than normal (n = 2) (Fig. 4F,H). Hence, reduced Six3 activity also interfered with OS morphogenesis.

To find whether OS cells of double mutants differentiated into glia of the optic nerve we generated an *rx3:Kaede* transgenic line, in which cells derived from the eye field, including OS and eye cells, express the photoconvertible protein Kaede^41^. When OS cells are photoconverted at 24–27 hpf in otherwise WT embryos and are imaged again at 48 hpf or later, photoconverted glial cells are clearly seen at the periphery of the optic nerve (n = 4) (Fig. 4I,K). By contrast, when the same experiment is performed in double mutants, only few or no photoconverted glial cells can be identified in the optic nerve (n = 3) (Fig. 4J,L), suggesting that when Six3 activity is reduced, OS fails to differentiate normally.

Together, the data show that OS morphogenesis and differentiation are abrogated in Six3-deficient embryos. The absence of a normal OS likely contributes to the observed RGC axon pathfinding errors.

### Reduced *cxcl12a* signalling from the optic stalk

OS cells normally express the chemokine ligand *cxcl12a*, which was shown to function in intra-retinal guidance of RGC axons by attracting them towards the OS^10^,^42^. The failure of OS differentiation in Six3-deficient embryos prompted us to examine *cxcl12a* expression at 30 hpf, when axons navigate within the retina. Indeed, *cxcl12a* expression in the OS was strongly reduced (Fig. 4M,N), providing a possible mechanism underlying intra-retinal pathfinding defects in *six3a;six3b* double mutants.

### Abnormal diencephalic midline formation in Six3-deficient embryos

**Figure 5:**
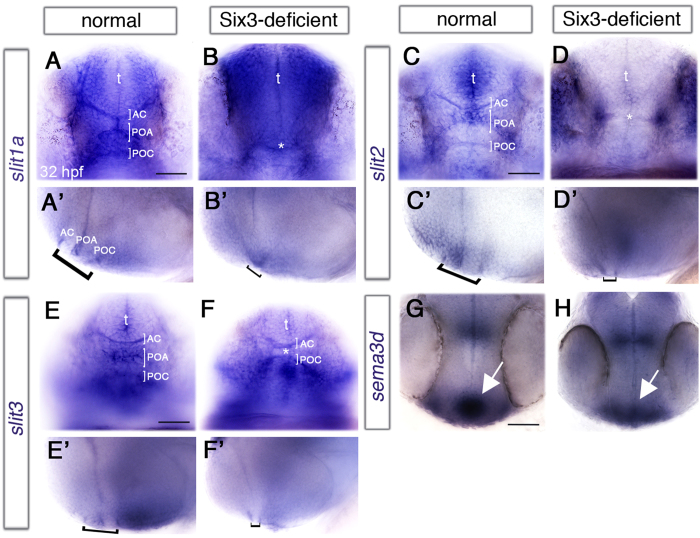
Abnormal midline signalling in Six3-deficient embryos. (**A–H**) Expression of signalling molecules in anterior diencephalic midline of normal (**A,C,E,G**) and Six3-deficient (**B,D,F,H**) 32 hpf embryos. (**A–F**) frontal views, (**A’–F’**) the same embryos as in (**A–F**) respectively, lateral views. (**A,A’,B,B’**) *slit1a* expression. Asterisk in (**B**) marks an apparently single commissure. (**C,C’,D,D’**) *slit2* expression. Asterisk in (**D**) marks what appears as a single commissure. (**E,E’,F,F’**) *slit3* expression. Asterisk in F marks the reduced POA. Brackets in all lateral views mark the POA flanked by AC and POC. (**G,H**) *sema3d* expression. Arrows point at the POA. AC, anterior commissure; POA, preoptic area; POC, post-optic commissure; t, telencephalon. (**G,H**) are dorsal views, anterior down. Anterior is to the left in lateral views. Scale bars are 50 μm.

After exiting the eye and OS, all RGC axons in zebrafish cross the anterior diencephalic midline, where they form the optic chiasm in close proximity to the diencephalic post-optic commissure (POC) and ventral to the telencephalic anterior commissure (AC)^43^,^44^,^45^. The region flanked by the AC and POC is the POA, in which expression of *semaphorin 3D* (*sema3d*) is required for midline crossing of RGC axons^46^. Additionally, correct expression of Slit family ligands *slit1a*, *slit2* and *slit3* in this region is required for normal commissure formation^45^. The failure to form a normal optic chiasm and the close proximity of the chiasm and commissures prompted us to examine expression of these signalling molecules in Six3-deficient embryos at 32 hpf, shortly before RGC axons normally approach the midline. We found that POA expression of *sema3d* and the three Slit-family genes was strongly reduced or missing (Fig. 5A–H). Moreover, where the AC and POC could be detected, they appeared much closer to each other than normal (Fig. 5B’).

**Figure 6:**
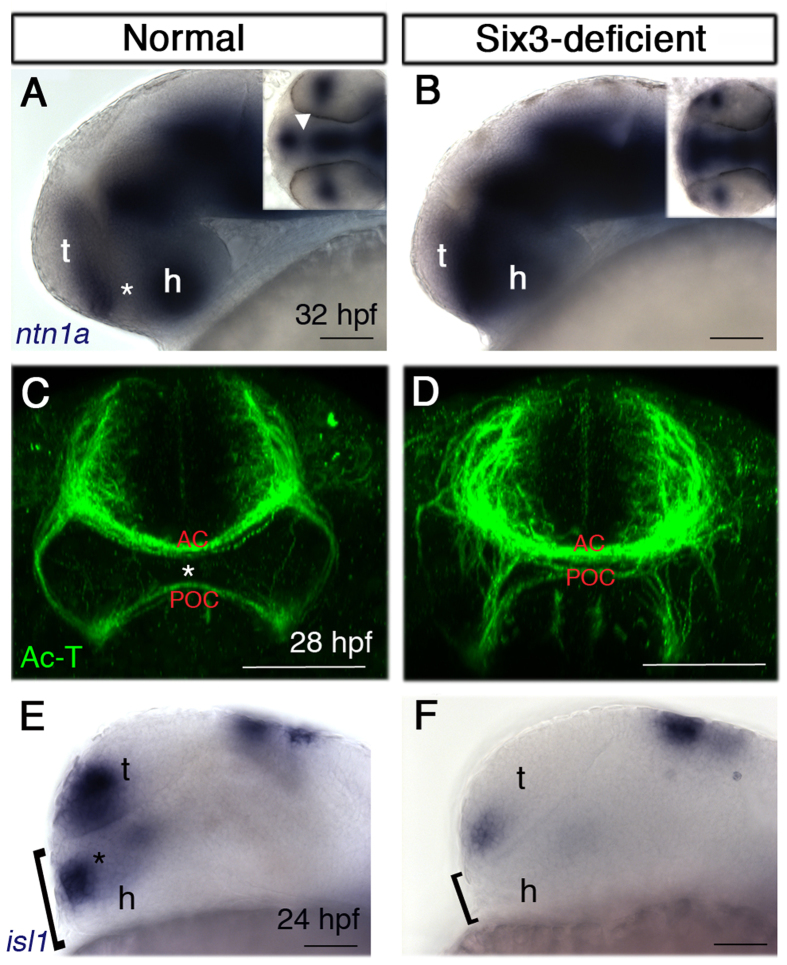
The POA is severely reduced in Six3-deficient embryos. (**A,B**) *ntn1a* expression at 32 hpf in normal (**A**) and Six3-deficient (**B**) embryos. Insets are dorsal views of the same embryos. Asterisk and arrowhead (inset) in A mark the *ntn1a*-negative POA. (**C,D**) Ac-T immunohistochemistry at 28 hpf in normal (**C**) and Six3-deficient (**D**) embryos. Asterisk in (**C**) marks the POA. (**E,F**) *isl1* expression at 24 hpf in normal (**E**) and Six3-deficient (**F**) embryos. Brackets mark the dorsoventral dimension ventral to the anteriormost telencephalon. AC, anterior commissure; POA, preoptic area; POC, post-optic commissure; t, telencephalon; h, hypothalamus. (**A,B,E,F**) are lateral views, anterior to the left; (**C,D**) are frontal views. Scale bars are 50 μm.

The abnormally close apposition of commissures raised the possibility that the POA was under-developed, providing an anatomic rather than regulatory explanation for the lack of signalling molecule expression. To test this hypothesis we examined expression of *netrin1a* (*ntn1a*), which is normally expressed at 32 hpf in the telencephalon and hypothalamus, leaving a gap in the POA (Fig. 6A). By contrast, in Six3-deficient embryos (n = 4) we observed a continuous expression domain throughout the anterior diencephlic midline and the telencephalic and hypothalamic domains appeared fused (Fig. 6B), supporting the idea that the POA was strongly reduced. Further confirmation for the reduction in POA was obtained by labelling the AC and POC at 28 hpf. In double mutant embryos (n = 3), the distance between the POC and AC was strongly reduced and commissures appeared fused together (Fig. 6C,D). We next labelled embryos for *islet1* (*isl1*), which is normally expressed in the POA at 24 hpf. In Six3-deficient embryos (n = 5), *isl1* POA expression could not be detected and importantly, the region ventral to the telencephalon appeared reduced at its dorsoventral dimension, suggesting that the POA was already under developed at 24 hpf (Fig. 6E,F).

These results demonstrate that in Six3-deficient embryos the diencephalic region where optic nerve axons form the chiasm is severely reduced, leading to diminished expression of signalling molecules and likely contributing to abnormalities in midline crossing of RGC axons.

### Delayed and abnormal differentiation of Six3-deficient RGCs

The abnormalities described so far in retinal, OS and POA development likely contribute non cell-autonomously to pathfinding errors of RGC axons. However, reduced Six3 levels might also cause abnormalities in RGCs themselves that would lead to abnormal optic nerve development. For example, intra-retinal navigation errors could arise from lack of RGC pioneers^11^ or loss of *cxcr4b* function in RGCs^10^, and navigation errors outside the retina are found when function of the Slit receptor Robo2 in RGCs is abrogated^6^,^9^.

**Figure 7:**
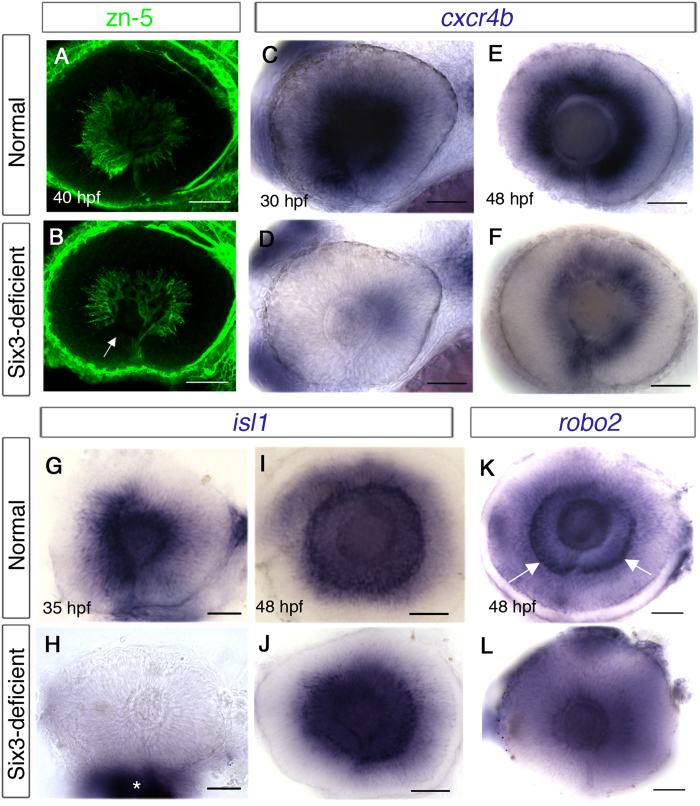
Delayed differentiation of RGCs in Six3-deficient retinas. (**A–L**) Eyes in whole embryos (**A–F**) or dissected eyes (**G–L**). (**A,B**) zn-5 immunohistochemistry of eyes in normal (**A**) and Six3-deficient embryos (**B**). Arrow in (**B**) points at the anterior-ventral region lacking differentiated RGCs. (**C–F**) WISH for *cxcr4b* at 30 hpf (**C,D**) and 48 hpf (**E,F**) in normal (**C,E**) and Six3-deficient (**D,F**) retinas. (**G–J**) WISH for *isl1* at 35 hpf (**G,H**) and 48 hpf (**I,J**) in normal (**G,I**) and Six3-deficient (**H,J**) retinas. Asterisk in H marks remnants of tissues outside the eye, which was dissected. (**K–L**) WISH for *robo2* at 48 hpf in normal (**K**) and Six3-deficient (**L**) retinas. Arrows in (**K,L**) point at RGCs that express *robo2*. All panels are lateral views, anterior to the left. Scale bars are 50 μm.

One line of evidence that RGC development is affected by Six3 loss of function comes from our earlier observation that ventroanterior RGCs, which are the first to differentiate, were not detected or appeared reduced in number in double mutants, at a time point when later-differentiating RGCs already sent axons (Figs 2B and 7B). As ventroanterior RGCs are part of the population of RGC pioneers whose axons guide later born RGC axons in the retina^11^, it is possible that their reduction/absence contributes to the phenotype of intra-retinal guidance errors.

We also examined retinal expression of chemokine receptor *cxcr4b*, which is required in RGCs for navigating towards the OS where its ligand, *cxcl12a* is expressed^10^. *cxcr4b* expression begins in a ventral patch at 24 hpf, before RGCs differentiate and continues in differentiated RGCs^47^. At 30 hpf, strong *cxcr4b* expression was present in RGCs of WT embryos, but was strongly reduced in Six3-deficient retinas (n = 4) (Fig. 7C,D). We considered two possible explanations for the lack of *cxcr4b* expression, namely specific downregulation in mutant RGCs or delayed differentiation of RGCs. To distinguish between these possibilities we labelled embryos for *cxcr4b* at 48 hpf and found that at this time point, Six3-deficient RGCs indeed expressed *cxcr4b* throughout the circumference of the eye, albeit at lower levels than normal (n = 3) (Fig. 7E,F), suggesting that reduced Six3 function caused delayed RGC differentiation. To verify this conclusion we labelled Six3-deficient embryos for *isl1*, which is normally expressed in differentiated RGCs^48^. Consistent with the idea of delayed differentiation, *isl1* expression was present in normal eyes and missing from eyes of double mutants at 34 hpf (n = 5) (Fig. 7G,H), but was present in double mutant eyes at 48 hpf (n = 6)(Fig. 7J). As *cxcr4b* is expressed in Six3-deficient RGCs, it is unlikely to play a significant role as a cell-autonomous factor in intra-retinal pathfinding in the double mutants. However, the results uncover a role for Six3 in regulating timely differentiation of RGCs.

Next, we asked if a cell-autonomous mechanism contributes to abnormal pathfinding errors outside the eyes of Six3-deficient embryos. As the pathfinding errors exhibited by Six3-deficient RGC axons are reminiscent of those seen in *astray* mutants^6^,^9^, we focused on *robo*2 expression. *robo2* is expressed in RGCs soon after their differentiation, beginning at 31 hpf, and more robustly by 36 hpf^9^. However, in Six3-deficient embryos *robo2* levels were strongly reduced even at 48 hpf, when many RGC axons have already reached the midline (Fig. 7K,L). Hence, Six3-deficient RGCs fail to activate expression of *robo2* on time and have reduced ability to respond to specific guidance cues in their environment.

Together, the results suggest that both cell-autonomous and non cell-autonomous mechanisms likely contribute to the pathfinding errors of Six3-deficient RGC axons.

## Discussion

In this work we present a new model of Six3 loss of function, in which reduction in total Six3 activity through inactivation of *six3a* and *six3b*, two out of three zebrafish *six3*-related genes (*six3a*, *six3b*, *six7*), results in malformed eyes and optic nerves. Because the eye malformations are relatively mild compared to previously described models of Six3 loss of function^16^,^18^,^21^, *six3a;six3b* double mutants provide an opportunity to study *in vivo* how Six3 functions during optic cup and neurogenesis stages, thus affording identification of previously unknown functions of Six3 in eye and optic nerve development.

The different combinations of double loss-of-function reveal the division of work between zebrafish *six3*-related genes. Although all three genes are expressed from early stages in similar patterns^20^,^49^, loss of *six3b* with downregulation of *six7* causes severe microphthalmia or anophthalmia through failure to form optic vesicles^21^ (our unpublished observations), whereas strong reduction of *six3a* function with loss of *six3b* function results in only mild microphthalmia and in eye abnormalities that are apparent at later stages. Labelling *six3a;six3b* double mutant embryos at early segmentation stages for eye field and anterior neural plate markers suggests that early patterning of the anterior neural plate is normal (Supplementary Fig. S4). Interestingly, combined loss of *six3a* and *six7* does not lead to overt eye phenotypes (A.S. and A.I., unpublished observations). Hence, *six3a* appears not to have a significant requirement during the transition from eye field to optic vesicle stage and to act redundantly with *six3b* only in specific aspects of eye and optic nerve development. This division of work between *six3*-related genes makes zebrafish a convenient system in which to dissect the roles of Six3 during different stages of eye formation. Interestingly, a previous study described the combined loss of *six3a* and *six3b* through the use of antisense morpholino oligonucleotides (MOs) against both genes^50^. This approach led to more significant microphthalmia than what we found, a difference that might stem from differences between the levels of loss of function achieved by mutation versus the use of MOs.

*six3a*;*six3b* mutants also uncover a specific role for Six3 in promoting development of the POA. The POA was recently shown to develop as a distinct morphogenetic entity, the optic recess region (ORR), which is organized around the optic recess of the third ventricle^51^. Our results suggest that development of this region is particularly sensitive to Six3 levels and further studies will elucidate the mechanisms through which Six3 promotes POA development.

Importantly, the ventral forebrain deficiencies and optic nerve hypoplasia found in *six3a;six3b* mutants place Six3 as a potential causative gene in additional congenital diseases involving the forebrain and eyes such as septo-optic dysplasia (SOD) and Kallman syndrome. Indeed, a mutation in *SIX3* was reported in a patient with Kallman syndrome^52^ and a connection to SOD is supported by the fact that Six3 expression is regulated by Sox2, a causative gene in SOD^53^,^54^,^55^.

We find that reduced levels of Six3 lead to a wide repertoire of RGC axon pathfinding errors. Intra-retinal pathfinding defects are relatively mild and are more prominent in the ventral retina. One possible mechanism is the failure of some ventroanterior pioneers to differentiate on time (Figs 2B and 7B)^11^. Another likely cause for the intra-retinal errors is the strongly reduced level of *cxcl12a* expression in the OS. However, the phenotype in Six3-deficient embryos is surprisingly mild when compared to reports on reduced Cxcl12a levels by morpholino or mutation^10^,^42^. One possible explanation is that residual *cxcl12a*, at levels undetected by ISH, is present in the OS, providing attraction for RGC axons.

Innervation of the ipsilateral tectum in the mutants appears to be mediated by several factors. Non-autonomous mechanisms include abnormal development of the OS and diencephalic midline, the latter resulting in reduced levels of *sema3d*. Interestingly, similar abnormalities in OS morphogenesis and differentiation were found when Pax2 function was lost^39^,^40^, suggesting Pax2 is a major mediator of Six3 function in OS development. A reduced POA was also reported in *Pax2* mouse knockout but not in zebrafish *pax2a* mutants^39^,^40^. Hence, this aspect of the phenotype is likely not mediated by reduced Pax2 function.

The possibility of a cell-autonomous mechanism that contributes to pathfinding errors outside of the eye is raised by the reduced level of *robo2* in Six3-deficient RGCs. Nevertheless, some phenotypes reported for *robo2* loss of function were not observed in *six3a;six3b* double mutants; this could be explained by low levels of *robo2* still present in Six3-deficient RGCs.

*six3a;six3b* double mutants uncover novel roles for Six3 in RGC differentiation. Firstly, there is delayed differentiation of RGCs and possibly other retinal cell types (A.I., T.A. and A.M.R., unpublished). This is likely mediated by regulation of cell cycle progression and/or exit. Indeed, Six3 has been shown to influence the cell cycle in other contexts, mostly during earlier stages of eye and anterior neural plate development^56^,^57^,^58^,^59^. Future analyses will determine the mechanisms by which Six3 influences timing of retinal differentiation.

A second effect of Six3 on RGC differentiation is abnormal gene expression in RGCs. In this work, for example, we found strongly reduced levels of *robo2* in RGCs at 48 hpf, which cannot be attributed solely to delayed differentiation as at this stage many mutant RGCs have axons that have reached the midline (Fig. 2D) and express the differentiation marker *isl1* (Fig. 7J). Hence, Six3 appears to influence RGC development through affecting specific gene expression. Additional roles for Six3 in differentiated RGCs are also likely, based on its expression in these cells after neurogenesis^25^,^27^, and it will be important to identify these functions and their contribution to RGC biology.

Many of the RGC axon pathfinding defects in Six3-deficient embryos are also seen in mutants in which Hh pathway activity is compromised^6^,^7^,^8^. A connection between Six3 and Hh signalling is of particular interest given that both have been implicated in HPE. In mice, Six3 was shown to be required for expression of Sonic hedgehog (Shh) in the anterior ventral diencephalic midline^60^,^61^, suggesting Six3 acts upstream of Hh signalling in patterning the anterior diencephalon. However, work in zebrafish showed that Six3 does not function upstream of Hh but rather in parallel in patterning the telencephalon^62^. In the current study, several observations are consistent with Six3 functioning upstream of Hh signalling, namely reduced levels of known Hh downstream targets such as *pax2a*, *cxcl12a* and *vax2*^38^,^42^,^63^. However, other findings argue against reduced Hh activity in *six3a;six3b* double mutants, and include lack of reduction in *ptch2* and *gli1* levels in the midline (A.S., A.M.R. and A.I., unpublished observations), no reduction in *vax1* levels in the OS (Fig. 3A,B) and no cyclopia, which would be expected if the deficiencies in midline development were mediated by reduced Hh signalling^38^,^45^. Interestingly, the enlarged optic stalk, coloboma and ectopic retinal tissue in the brain are more reminiscent of *patched2* mutants, in which Hh pathway activity is upregulated^64^. However, molecular changes found in *ptch2* mutants such as expanded *pax2* expression and increased Hh signalling are also inconsistent with our findings in Six3-deficient embryos. Hence, based on current findings we propose that Six3’s function in optic nerve development is not mediated by regulation of Hh pathway activity.

Interesting similarities also exist between phenotypes observed in Six3–deficient embryos and *lhx2b* (*belladonna*) mutant embryos. For example, in *lhx2b* mutants RGC axons typically fail to cross the midline and POA development appears reduced^6^,^12^. These similarities are particularly interesting given that *lhx2b* has been proposed to function as a mediator of Six3 function during early eye and forebrain growth^50^. However, in *lhx2b* mutants AC and POC fail to form unlike in Six3-deficient embryos and we did not observe significant reduction in *lhx2b* levels in Six3-deficient embryos (A.M.R. and A. I., unpublished). Hence, while it is still possible that *lhx2b* functions downstream of Six3 in forebrain patterning and RGC axon guidance, it does not appear to be a significant mediator of Six3 activity in this context.

## Methods

### Fish lines and genotyping

*six3a*^*vu129*^ mutation was identified by TILLING (Targeting Induced Local Lesions IN Genomes) as previously described^28^,^29^. *six3b*^*vu87*^mutation has been described^21^. *six3b*^*vu87*^ and *six3a*^*vu129*^ fish were maintained in AB and TL backgrounds. Genotyping of *six3a*^*vu129*^: a 176 bp fragment was amplified from *six3a* gene using forward primer 5′- AGTTTCCCCTGCCTAGAACC-3′ and reverse primer 5′- AAACCAATTTCCGACCTGTG-3′. After digestion with AleI, the mutant allele is cut into 80 bp and 96 bp fragments whereas the wild-type (WT) allele remains uncut. *six3b*^*vu87*^ was genotyped by amplifying a 168 bp fragment using forward primer 5′-TCAACAAGCACGAGTCCATC-3′ and reverse primer 5′-GCAGCTTCTCTGCTTCTTGG. After digestion with MseI, the mutant allele is cut into 65 bp and 103 bp fragments whereas the WT allele remains uncut. Generation of *Tg(rx3:Kaede)huj8* line: Kaede coding sequence from pKaede-S1 (Amalgaam) was cloned into pENTR1A (Invitrogen) to generate pME-Kaede, which was used for inserting Kaede sequence by Gateway LR recombination downstream of *rx3* promoter sequence in *rx3*-destination plasmid^65^. *Tg(rx3:Kaede)* fish were generated by co-injection of *rx3:Kaede* DNA and Tol2 transposase synthetic RNA as described (Kawakami *et al*. 2004). Founder fish were identified by transgene expression in their progeny.

All experiments were approved by the Hebrew University Authority for Biological and Biomedical Models. The methods were carried out in accordance with the approved guidelines.

### RNA injection

pCS2-*six3a*: *six3a* coding sequence was PCR-amplified and cloned into BamHI and XbaI sites of pCS2+ vector. pCS2-*six3a-L183S*: L183S mutation was introduced by site directed mutagenesis into pCS2-*six3a*. Sequence was confirmed for both constructs and synthetic capped RNA was prepared by NotI digestion and transcription with SP6 polymerase (mMESSAGE mMACHINE, Ambion).

### Visual motor response (VMR) assay

Automatic quantification of larval movement was performed as previously described^36^. Briefly, at 6 dpf, *six3a;six3b* double-mutant and WT larvae were placed, individually, in 48-well plates in an observation chamber of DanioVision Tracking System (Noldus Information Technology, Waningen, Netherland). After 3 hours of light adaptation, larvae were subjected to 4 intervals of 30 min light/30 min darkness. In all experiments, larvae were subjected to locomotion analyses between 12:00 to 4:00 PM in a sound- and temperature-controlled (26 °C) behavioral testing room. Locomotor activity was tracked and analyzed using Ethovision 8.0 software (Noldus Information Technology). Described results are combined from two independent experiments.

### *In situ* hybridization, immunohistochemistry and histology

Whole-mount *in situ* hybridization (WISH) using riboprobes was performed according to standard protocols^66^. BM Purple (Roche) was used as color substrate.

Whole-mount immunohistochemistry (IHC) was performed as previously described^65^. The following antibodies and dilution were used: mouse anti-acetylated α-tubulin (1:1,000; clone 6-11B-1, Sigma-Aldrich), mouse anti-zn-5 (1:100; ZIRC), Alexa Fluor 647-conjugated [F(ab’)_2_ fragment] donkey anti-mouse (1:500; Jackson ImmunoResearch).

For histology, embryos were fixed in 4% paraformaldehyde (PFA) overnight at 4 °C, washed with PBT, dehydrated in EtOH series and embedded in JB4 resin (Polysciences, Inc.) according to manufacturer’s instructions. 4 μm sections were cut with LKB8800 Ultratome III microtome and stained with methylene blue—azure II.

### Lipophilic dye labelling

For anterograde labelling of retinotectal projections, embryos were fixed in 4% PFA overnight at 4 °C, washed with PBS, bleached in 3% H_2_O_2_, 1% KOH (1M) in PBS for 2 hrs or more to remove eye pigmentation and rinsed several times with PBS. Embryos were mounted in 1.2% low-melting-point agarose gel and DiI (1, 1′-dioctadecyl-3, 3, 3′, 3,-tetramethyl indocarbocyanine perchlorate, Sigma-Aldrich #468495) or DiO (3,3′-dioctadecyl oxacarbocyanine perchlorate; Sigma-Aldrich #D4292) was applied either by injection of dissolved dye in 1% chloroform between lens and GCL or by direct application using a dye-coated insect micro pin (Ø 0.1 mm, FST #26002-10). In both methodologies, specimens were kept overnight at 28 °C to allow dyes to diffuse along the axons before imaging.

### Photoconversion and imaging

To block pigmentation when imaging embryos older than 24 hpf, embryos were raised from 22 hpf in the presence of 0.003% N-Phenylthiourea (PTU; Sigma-Aldrich #P7629). Live embryos were anaesthetized using tricaine and mounted in 0.5% low-melting-point agarose (SeaPlaque, Lonza). For photoconversion, images of eye and OS region were acquired by minimal exposure using a 488 nm laser. A selected region of interest (ROI) was exposed to 405 nm laser scanning until all green fluorescence was converted to red, as determined by imaging with 488 nm and 555 nm lasers. The embryos were released from agarose and incubated at 28.5 °C in the dark until imaged again.

Images were acquired using Zeiss LSM700 confocal microscope and Axio Imager.M2 compound microscope or with Discovery.V8 stereoscope and AxioCam MRc digital camera (Zeiss). Microscope objective used were 40 × 1.0 NA water objective or 25 × 0.8 NA or 10 × 0.3 NA. Images were exported as JPEG or TIFF files using ZEN 2009 LE software (Zeiss) and figures were assembled using Adobe Photoshop CS4 software.

## Additional Information

**How to cite this article**: Samuel, A. *et al*. Six3 regulates optic nerve development via multiple mechanisms. *Sci. Rep*. **6**, 20267; doi: 10.1038/srep20267 (2016).

## Supplementary Material

Supplementary Information
