# Supplementary material for: Six3 regulates optic nerve development via multiple mechanisms
Source: Sci Rep. 2016 Jan 29;6:20267. doi: 10.1038/srep20267 (PMC4731751; doi:10.1038/srep20267)
Supplement: Supplementary Information [file srep20267-s1.pdf]

Supplementary Information to:

Six3 regulates optic nerve development via multiple mechanisms

Anat Samuel[1], Ariel M. Rubinstein[1], Tehila T. Azar[1], Zohar Ben-Moshe[2],

Seok-Hyung Kim[3,4] and Adi Inbal[1,*]

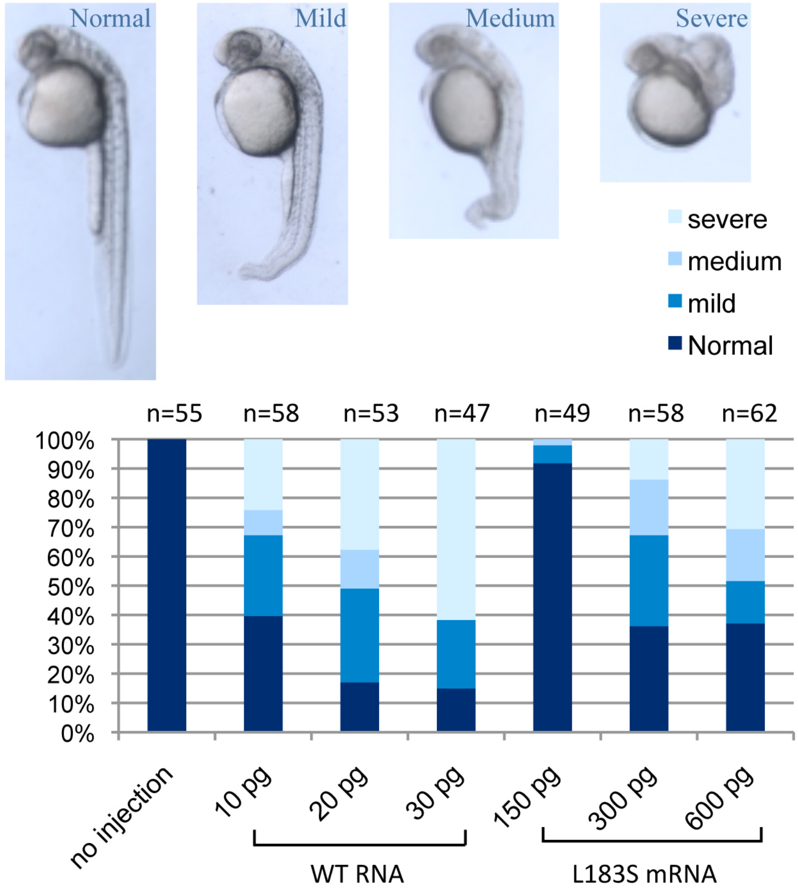

Figure S1. Results of normal *six3a* and *six3a*^{vu129} misexpression. Live embryos at 26 hpf after injection of synthetic RNA encoding normal or mutant Six3a^{L183S}. Four phenotypic groups were scored: normal looking, mild: slightly dorsalized, medium: moderately dorsalized and severe: strongly dorsalized. The percentage of embryos from each group after injection of different doses of normal or mutant *six3a* RNA is shown in the graph, with phenotypes color-coded. The number (n) of embryos in each group and injection doses are depicted on top and bottom of bars, respectively.

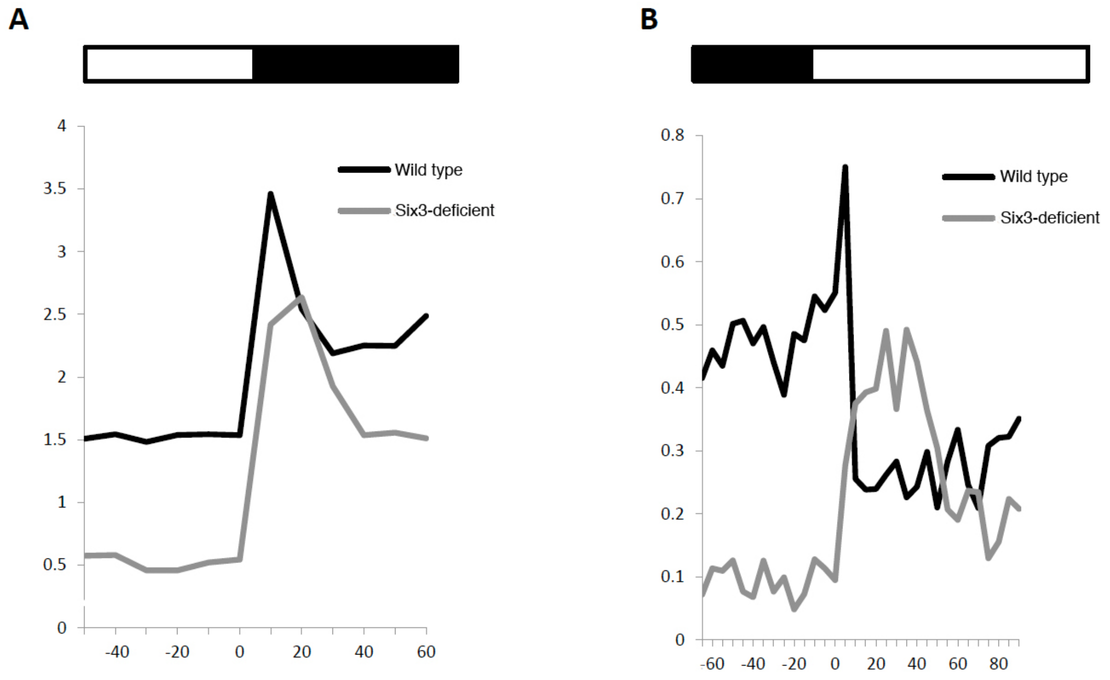

Figure S2. Visual-motor responses of Six3-deficient larvae. (A,B) Locomotor behavior of 6 dpf WT and Six3-deficient larvae expressed in cm per 10 seconds (A) or 5 seconds (B). Each trace represents an average from four events, of 80 responses from 20 individual larvae.

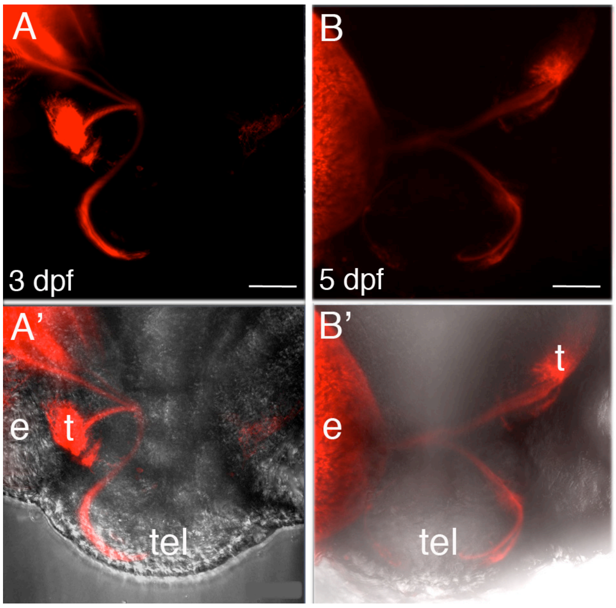

Figure S3. RGC axon misrouting in Six3-deficient embryos. (A, B) Two examples from 3 and 5 dpf Six3-deficient embryos, of RGC axon misrouting with axons projecting from one eye both to tectum and telencephalon. (A', B') are the same images as A and B, respectively, with overlay on bright field image to help visualize anatomy. Eyes were labelled with DiI. e, eye; t, tectum; tel, telencephalon.

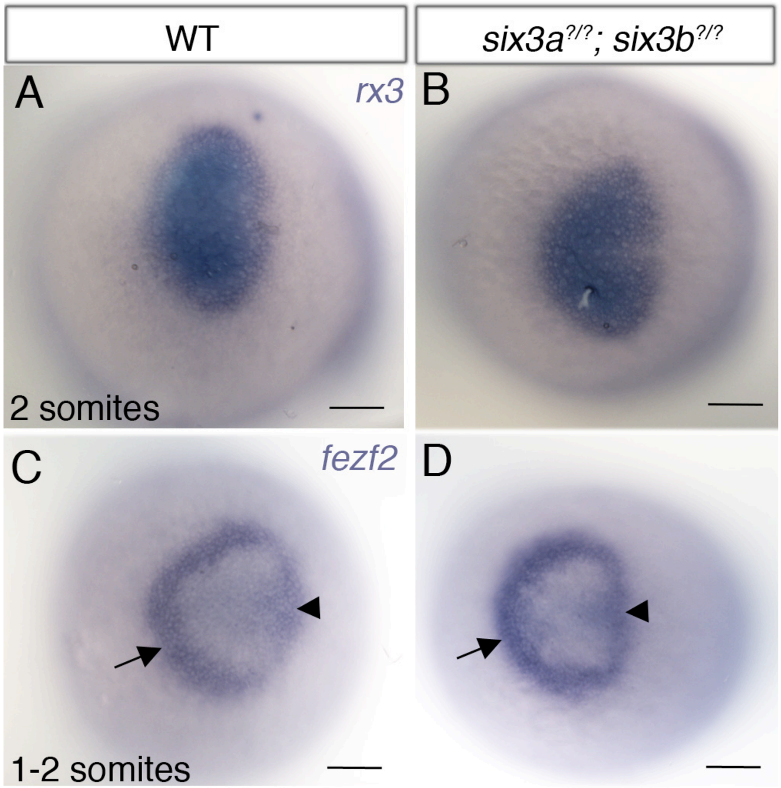

Figure S4. Early neural plate patterning appears normal in *six3a;six3b* double mutants. (A-D) Wild-type embryos (A,C) and embryos from crosses between *six3a;six3b* double heterozygous parents (n>100 for each labelling) (B,D) were labelled by *in situ* hybridization for eye field marker *rx3* (A,B) and neural plate marker *fezf2* (C,D) at early segmentation. All embryos in clutches from *six3a;six3b* double heterozygous parent appeared similar and comparable to wild type. (C,D) Arrows and arrowheads point at presumptive telencephalon and ventral diencephalon, respectively.
